# Uterine Therapeutics

**Published:** 1884-03

**Authors:** P. P. Wells


### BOOK NOTICES.

UTERINE THERAPEUTICS. By H. Minton, A. M., M. D. New York: A. L. Chatterton Publishing Company. 1884.

We welcome the appearance of this volume of seven hundred and ten pages, not only as the outcome of years of diligent endeavor to furnish aid to faithful workers in their endeavors as healers in the department of therapeutics to which this volume is devoted, but as a timely and not too early witness to the truth that much of the manual and mechanical interference with uterine ailments, which passes at present under the rather imposing term of "Gynæcological Surgery," is wholly unnecessary, and not unfrequently injurious, morally and physically. That most of these ailments are better and more successfully treated by the use of the truly related homœopathic remedy than by any mechanical interferences or appliances has for a long time been with us an earnest conviction, fully sustained by our own not very limited experience, now extending over forty-five years, in which we have *in no one case* had recourse to any mechanical contrivance whatever. In our hands the specific remedy, when found, has left us and our patients with no need of help from any other source.

That there have been difficulties in finding the remedy in many cases is true enough. But diligence and perseverance has enabled us to secure to our patients the above stated results. The difficulty has been partly in the nature of the cases treated, many of which express but obscurely symptoms which are most important to specific therapeutics, and partly from defect of our materia medica, growing out of the fact that so large a majority of the provers from whom we have our record were males, and could, therefore, give us nothing as to the effects of the drugs proved on the female organization. This difficulty has been to some extent lessened by the contributions of those women who have been admitted to our ranks as colaborers of late years.

The difficulty, that of finding the specific for any given case, has been reduced to a minimum by the zeal, diligence, perseverance, and intelligence of our excellent neighbor and friend, the author of the volume before us. A book with a plan and a purpose, the plan being clearly adapted to the end in view and the purpose a laudable one, is a book always to be thankfully accepted from the hands of the author, and all the more when it treats of a subject so important as that to which the volume before us is devoted. Its plan is excellent, being so like that of the peerless Bœnninghausen in his *Therapeutics of Fever* as to suggest its having been modeled on that peerless work. First, so to say, a group of the concrete symptoms of the medicines brought within its scope, and then a repertory, giving in detail the symptoms with the needful concomitants and modalities, in such fullness as to reduce the finding of the required remedy to a minimum of difficulty, except to such as are embarrassed by a wealth of means or confused by a "wilderness of symptoms." We have no hesitation in commending the volume to all lovers of truth and decency and all who are workers under the guidance of our law as healers, and especially those who are more interested in their labors for the relief of the class of sufferers for whose benefit the work has been prepared.

The purpose of the book, the motive of its author in its preparation, was, no doubt, to lighten the labors of others in a department of work which he had long, faithfully, and, we have no doubt, successfully pursued. We do not know if beyond this he aimed a blow at what has come to be one of our greatest modern abominations. It cannot be truthfully denied that what now passes and is now practiced as *Gynæcological Surgery* has come to be a source of demoralization often to patient and practitioner of the gravest character. We do not know if it was the object of the author to silently rebuke this abomination, but we do know that a faithful use of the means his book puts into the hands of the profession will, in the assured successes which will result, successes without offense to delicacy or moral stain, to practitioner or patient, will multiply rebukes of the prevailing abominations in every cure so wrought, by adherence to pure law and its corollaries.

It will not be understood by the above that we suppose our author opposed to, or to be undervaluing, any right and proper dealing with cases which are proper subjects of surgical interference. Neither are we. But the craze to which we refer makes no discrimination. It assumes uterine or ovarian affection, proceeds to search for it, and as the search is good for nothing unless something is found, and if nothing is found the practitioner is in danger of discredit, therefore something *is* found, and this something is asserted to require further instrumental or mechanical interference, and this implies further exposures and advancing demoralization, and this, too often, to end in new and additional troubles, demonstrating the worthlessness, to say the least, of all this parade of that which may have had in it little of knowledge or skill.

This sketch is from life, as the following cases will demonstrate. A patient of the writer, married late in life, after the severe labor at the birth of her first child had occasional slight attacks of leucorrhœa with pains in the sacral regions. These were promptly relieved by medication. Their return impressed the patient with fear of cancer. Though told she had no symptoms of this dread plague, she listened to the advice of one of those kind lady friends, who know all about it, and consulted one of those doctors who "make female complaints a specialty." The doctor introduced the speculum, and, of course, found what was sought. "What!" was the exclamation. "No cancers! Why, here are *six!*" That was enough to satisfy the patient that she had found the right doctor at last. This discovery was followed by frequent subsequent use of speculum and local applications, till the doctor said "*five of them were healed.*" But the sixth and the largest was "higher up," and now this was to be attacked direct. The endeavor, in accord with this diagnosis, to introduce the medicated application into the interior of the uterus was followed by an intense metritis, from which the patient escaped with life but not with confidence in the knowledge or skill of her before admired doctor. The cancers were suspected, and, therefore, plenty of cancers were to be found, and they were. It was only when the doctor would give the *coup de grace* to the biggest of them by one grand effort of genius and skill that the whole imposture was apparent to patient and friends.

A second and different case which ultimately came under our care for repair of damages well illustrates another form of the evil we deprecate. Miss ——— was a young lady of twenty years, far above the average of intelligence and refined sensibility, of a family numbered in the first social circles of our city, and she sought the advice of a doctor for some trifling ailment. This was at a time when the speculum craze was young, and the doctor had a speculum, and he persuaded his patient that it should be used; and he used it. Then, of course, he must appear to be up in all which then and now goes to make up "*local treatment.*" He had to find *something* by the specular examination or what was the use of it? It was found and treated by frequent subsequent use of the speculum and "local applications," with, as the only result, an irritation and inflammation of the ovaries which made her life a misery. After enduring this for some months, and resorting to *change* to country air for relief, she became my patient. The most careful examination of the case disclosed absolutely nothing in the past calling for the use of the curse, the speculum, or for "local treatment," and nothing appeared which was not reasonably and legitimately traced to this treatment as its origin. The patient was cured of all after some difficulty and is now an ornament of the society in which she moves.

This whole proceeding we do not hesitate to say is unnecessary to any best and *most* "*scientific*" efforts to relieve the troubles which have led to this unjustifiable proceeding. These troubles, those of them which admit of cure, are far more amenable to their specific remedies than to any mechanical means whatever. And the profession and the public are under no small obligation to our author for this contribution of his knowledge and labor to lessen the difficulties of the one, and give hope and help to the other in their sufferings from a class of evils altogether too common.

As to the manner in which our author has performed his task. The examination we have been able to give his book up to this present writing has disclosed nothing to be changed. Under some of the rubrics omissions have been found of remedies the experience of the writer would have placed there. These have been few. The whole work shows thoroughness which is as truly gratifying as is the work itself full of promise of usefulness. We wish it and its author the success they both merit. P. P. WELLS.

